# Immune dysregulation in patients with TRNT1 deficiency

**DOI:** 10.1186/1546-0096-13-S1-O88

**Published:** 2015-09-28

**Authors:** A Giannelou, Q Zhou, M Stoffels, D Stone, A Ombrello, K Barron, H Su, K Risma, L Sramkova, A Sediva, S Joshi, A Al Sonbul, H-W Sun, M Quezado, M Gadina, I Aksentijevich, DL Kastner

**Affiliations:** 1 National Institutes of Health, Bethesda, MD, USA; 2 Cincinnati Children's Hospital Medical Center, Cincinnati, OH, USA; 3 Motol University Hospital, Prague, Czech Republic; 4 Nationwide Children's Hospital, Columbus, OH, USA; 5 King Faisal Specialist Hospital & Research Center, Riyadh, Saudi Arabia

## Introduction

Next-generation sequencing has led to the discovery of a number of disease-associated genes in patients with uncharacterized autoinflammatory diseases and has pointed to new pathways regulating immune function.

## Objective

To investigate the pathogenesis of disease in patients with the deficiency of the CCA-adding enzyme tRNA nucleotidyltransferase 1 (TRNT1). The TRNT1 enzyme catalyzes an essential step for tRNA maturation and protein synthesis, however it is largely unknown how abnormalities in this pathway lead to inflammation and immunodeficiency.

Patients and Methods: Whole exome sequencing (WES) was performed in a consanguineous Saudi family with 2 affected siblings and in a parent-child trio; candidate gene screening was subsequently performed in 3 sporadic Caucasian patients. Cytokine profiling, tissue immunohistochemistry, and deep RNA and tRNA sequencing were performed in patients’ primary cells. Protein function was studied in zebrafish embryos.

## Results

We have identified 7 patients with biallelic homozygous or compound heterozygous mutations in the *TRNT1* gene. Three patients died due to multiorgan failure. We identified 6 disease-associated missense mutations that affect evolutionarily conserved amino acid residues. These variants are either novel or found at an allele frequency less than 0.0001 in public databases, consistent with recessive disease inheritance. Two apparently unrelated patients shared the same genotype. Recently, loss-of-function mutations in the same gene were reported in patients with a syndrome termed SIFD (sideroblastic anemia, immunodeficiency, periodic fevers and developmental delay). Four mutations from our study have not been reported in patients with SIFD. Knockdown of the zebrafish *TRNT1* homologue caused abnormalities resembling the human phenotype. Preliminary results of next-generation tRNA sequencing showed a significant down-regulation of mature tRNAs in patient's fibroblasts compared to healthy control. RNA-sequencing of patients’ whole blood showed an up-regulation in the expression of neutrophil-related genes. Consistent with these data, analysis of lesional biopsies from one patient's colon showed cryptitis with neutrophilic infiltration. Cytokine profiling of stimulated patients’ leukocytes and serum samples suggest that the inflammatory phenotype is likely driven by IL-6 and interferon. Treatment with TNF inhibitors has shown promising results in attenuating the systemic inflammation and stabilizing the anemia in 3 patients who experienced recurrent fevers and required transfusions.

## Conclusions

Hypomorphic mutations in *TRNT1* are associated with a new autoinflammatory disease manifesting a variable phenotype of fevers, congenital sideroblastic anemia, immunodeficiency, and developmental delay. Study of the underlying disease mechanisms might lead to the discovery of a new pathway regulating immune function and inflammation.

